# An energy- aware algorithm for optimizing dynamic zone monitoring and multiple cluster head selection with routing in border surveillance WSNs

**DOI:** 10.1371/journal.pone.0354345

**Published:** 2026-07-23

**Authors:** Jeevarathinam Jayachandran, Krishnasamy Vimala Devi

**Affiliations:** School of Computer Science and Engineering, Vellore Institute of Technology, Vellore, TamilNadu, India; Maha Bharathi Engineering College, INDIA

## Abstract

Border surveillance plays a vital role in national security, necessitating the deployment of efficient and strong Wireless Sensor Networks (WSNs) to reveal and protect sensitive areas. This research describes a unique approach for optimizing border surveillance that combines a hybrid energy efficient zone-based dynamic clustering method with routing utilizing Geometric Modified Ant colony Harris Hawk Optimization Algorithm (EECR-GeMACHHOA). Our proposed method aims to enhance energy efficiency and prolong the network lifespan. This technique starts with the identification of zone monitors using geometric translational symmetry capabilities, which enables the formation of zones. These zones are then optimized by making use of modified ant colony based on dynamic pheromone evaporation for multiple cluster head selection. The EECR-GeMACHHOA improves routing pathways by integrating geometric, nature-inspired Ant colony and Harris Hawk optimization methodologies in order to decrease energy utilization and maximize the packet transmission rate. The proposed approach reduces network latency by 6.66%, consumes 15.03% less energy, and sends 9.86% increase in packets sent to the base station relative to alternative methods. The outcomes demonstrate a very good increase in network lifetime, robust energy efficient system, and data packet transmission dependability when compared to contemporary strategies.

## Introduction

Detecting and preventing potential threats in border areas is critical for securing a nation. This means that advanced surveillance systems must be installed along the country’s borders. WSNs are a good fit because they can automatically monitor vast isolated places and engage in real-time data transmission. However, successful operation of these WSNs is challenging in several ways when they are deployed in border areas. With ever-growing technological advancements in the field of information technology, WSNs have created new possibilities as sensor nodes become more intelligent, wireless, and smaller. Sensor nodes have their own limitations which is limited battery capacity, that directly impacts a network’s lifespan, even though they are efficient in terms of data processing, data transfer, Anti-interference properties, low cost, and simplicity of installation. Fundamentally, wireless sensors are employed to gather information, track the network environment, and transmit the information to the lowest-level external sink node, or Base Station (BS) [1,2]. WSN are useful for many applications, including seismic monitoring, earthquake detection, aviation buildings, volcanic eruptions, cracks in buildings and ships, nuclear threat detection and radiation level measurement, and biological applications. Since the sensor nodes run on batteries, they are not replaceable, especially if they are situated in isolated locations like mountains, forests, or deserts. Battery energy must be utilized for the sensor nodes and sink node to efficiently distribute data packets. Consequently, enhancing the energy efficiency of sensor nodes is crucial for optimizing the overall performance of a network. Numerous studies have focused on energy conservation strategies for sensor nodes to prolong both node lifespan and network longevity [3].

**Fig 1:**
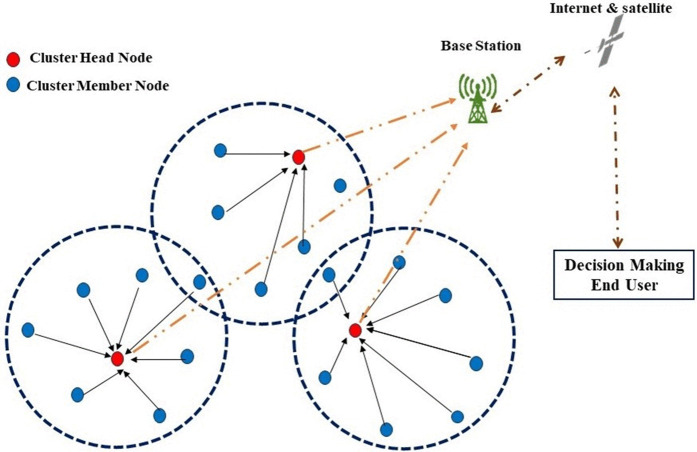
Structure of cluster-based sensor network.

Figure 1 depicts the fundamental topology of a cluster-based sensor network. Clustering is a widely used method for establishing WSNs, where sensor nodes are organized into groups called clusters. Each cluster is managed by a cluster head (CH), responsible for communication with member nodes, collecting data from them, and relaying it to the BS. Furthermore, CHs play a critical role in maintaining energy balance within the network, which helps prolong network lifespan and enhances the longevity of individual nodes. Clustering offers more benefits to WSNs in terms of energy balance with special characteristics: 1) The overall capacity of the network is preserved as the CH manages communication exclusively among the cluster members. (2) When the CH aggregates and sends the collected data to the BS, it helps conserve the energy of the member nodes, and (3) CH always keeps the local route configuration updated for the CH of other clusters, greatly enhancing network scalability [4].

CH selection is highly important in network optimization, and the residual power and distance between members and node degrees are the most important parameters that need to be considered when electing a CH. When the network scalability increases, the CH must adopt multi-hop communication to minimize energy usage. In such a scenario, the cluster near the BS receives heavy traffic information from other clusters; such a case is called a hotspot problem. In such a scenario, the BS is disconnected from the network; to address this issue, offering mobility to the sink node is a promising idea. A WSN has two phases when sensor nodes retrieve data, namely, intra and inter-cluster transmission, local CH participates in intra-cluster transmission by receiving data from its cluster members based on Time Division Multiple Access (TDMA) schedule; in inter-cluster transmission, on the other hand, a CH combines the data that it has received from its local members and sends it to BS. TDMA scheduling prevents nodes from overhearing and idle listening to conserve energy (intra-cluster), and CH data aggregation minimizes the overall amount of data sent to BS (inter-cluster). The cluster members with no forward data will be kept in idle or sleep mode to conserve their battery energy [5,6].

In existing clustering-based approaches CH is the main node that retains more responsibilities than the members of the cluster, so the CH’s load needs to be balanced in terms of data aggregation, intra-cluster communication, and transmission to the BS to increase its lifetime. Thus uneven workload often accelerates the energy depletion of CHs, thereby reducing overall network lifetime. To mitigate this, earlier studies have proposed load-balancing techniques, with periodic global re-clustering being the most common solution. However, such strategies introduce excessive communication overhead between CHs, cluster members, and the BS, ultimately shortening both CH and network lifetimes. Prior research has also shown that the cost of energy loss due to CH overload and the re-clustering process itself are comparable, making it essential to minimize simultaneously. Unlike these conventional methods, the proposed EECR-GeMACHHOA approach integrates the geometric approach for Zone Monitor (ZM) selection, modified ant colony approach for multiple CH selection and HHO optimization for energy-aware routing, thereby reducing re-clustering frequency, communication overhead, and premature CH failures. This highlights a key distinction from existing research, as our approach minimizes re-clustering overhead and energy loss but also ensures balanced load distribution among CH and ZM thereby extending the network lifetime.

### Problem attribution and motivation

According to this study, researchers understood that traditional clustering and routing methods were often limited by their high energy consumption, inefficient load balance, and adaptiveness to dynamic network conditions. The algorithms inspired by nature and meta-heuristic achieves better performance by combining various appropriate clustering methods. The lack of robust mechanisms to optimize cluster formation and secure routing while maintaining low energy usage motivates the development of advanced solutions. Selecting the optimal CH and preventing it from being overloaded when the amount of data traffic is high or the node density is large should be considered vital because it can directly affect the life span of nodes and network. Border surveillance face significant challenges, including energy constraints, scalability issues, which compromise their reliability and operational longevity. Existing methods often struggle to balance energy efficiency, and real-time adaptability in dynamic and heterogeneous environments. Furthermore, to minimize the CH workload, ZM can include a variable number of clusters per zone, but each cluster should have a uniform distribution of cluster members. In such scenarios, both single and multi-hop transmission need to be adopted, depending on the remaining residual power of the CH, and re-clustering needs to be performed to improve the data aggregation and prevent nodes from dying. Thus, there is still opportunity for development in terms of prolonging both node and network lifetimes and efficient data transmission with improved energy efficiency by adopting nature-inspired or metaheuristic algorithms along with better routing with elegant routing algorithms.

Based on the above discussions, the proposed method addresses these gaps by relying on the EECR-GeMACHHOA algorithm, a framework for energy-efficient hybrid routing called the hybrid zone method. This methodology improves energy efficiency and optimizes routing and ensures reliable and robust operation in the complexities of monitoring scenarios. The key contributions are as follows:

1. An adaptive geometric translational symmetry feature for selecting the ZM for each zone is proposed, and the threshold energy is defined for each zone. This guarantees balanced energy distribution during the network.
2. Proposed an adaptive clustering mechanism using modified ant colony optimization algorithm with dynamic pheromone evaporation for multiple CH selection for efficient data transfer to reduce the node’s energy usage between the CH and ZM and between the CH and CM. The CH selection considers the transmission range, node connectivity, node degree, average distance, highest residual energy, and precise parameters defining the threshold strength for multiple CH selection. To achieve network lifetime maximization, the ZM and CH undergo periodic changes for dynamic optimization.
3. The integration of HHO energy routing approach allows data transmission across the network both between clusters and between clusters to discover optimal, energy-efficient and low latency routing routes from ZMs to BS and ensure robust and scalable data transmission.
4. This algorithm is resilient, detecting the best path quickly if the link between the nodes is damaged, reduces latency, and increases reliability.
5. To demonstrate the superiority of the proposed method, its performance is assessed and its results contrast with those of competing strategies.

Adaptive clustering and routing algorithms using modified ant colony optimization (ACO) and HHO algorithms significantly improve network scalability, efficiency, and robustness, ensuring dynamic environment adaptation in real time. The proposed method uses energy balancing techniques by ZM, periodic updates to CH and adaptive clustering to improve energy use and extend the entire network life. Unlike traditional static environments, this work addresses the challenges of large-scale dynamic WSNs to ensure efficient data transmission, load balance, and scalability under different network conditions. Collectively, these contributions provide solid solutions for optimizing WSN deployment, energy efficiency and adaptability in a dynamic environment in the real world.

The organization of this article is outlined as follows. The related work presents the performance, limitations, and challenges of existing approaches. The proposed EECR-GeMACHHOA zone-based hybrid method introduces the network model, schematic design, flow diagram, multiple CH selection, and ZM selection strategies. The experimental results of the proposed approach evaluate it across a wide range of performance indicators. The statistical performance analysis reports the mean, standard deviation, and confidence intervals for key metrics and compares them against competing methods. An in-depth discussion of the results interprets the improvements achieved by the proposed approach. Finally, the article concludes and outlines potential directions for future research.

## Related work

The sturdiness of a WSN in most cases induced by means of key parameters, extensively the time till the primary node’s energy is exhausted. Application unique factors additionally play a massive position. A wealth of research emphasizes the significance of maximum appropriate CH preference for extending WSN lifespan. Recently, interest has centered on two predominant categories of strategies: optimization-based, and clustering-based techniques. These techniques often leverage strategies at the side of fuzzy logic, Genetic Algorithms, and diverse meta-heuristic optimization algorithms. This phase reviews modern-day advancements in these techniques, exploring their impact on improving WSN performance and prolonging network lifetime.

The authors of [7] introduced a centralized energy optimized protocol comprising two main components. First, load-balancing sequential algorithms were implemented to preserve the energy of CHs during inter-cluster clustering. Second, an intra-cluster clustering approach utilizing CH rotation is employed to evenly distribute energy utilization among nodes within each cluster. The network is divided into smaller clusters using a size-based clustering method, and a minimal number of cluster members are reassigned through inter-cluster energy adjustments to reduce overhead. The energy of inter-cluster communication was balanced by the usage of energy-aware data forwarding. The simulation results were compared with the network longevity, energy consumption, and throughput of MH-LEACH, LAR-CH, and EAHA. The suggested strategy has outperformed the other three techniques taken into consideration in this work. The authors of [8] presented a metaheuristic hybrid strategy that combined the use of Particle Swarm Optimization (PSO) and Genetic Algorithms (GA) to discover an optimal route for sink mobility-based data transmission, while GA was employed for CH selection. The outcomes of the simulation is evaluated against the latest methods based on throughput, stability, dead nodes, network lifespan, and remaining residual power in the network. The proposed GAPSO-H method demonstrated superior performance compared to other approaches, showing significant improvements across all metrics analyzed in this study. To alter the operating mode of sensor nodes, authors in [9] used GA in conjunction with a Selective Adjustment Mechanism that is primarily composed of two components: (1) increasing the working time during low-power consumption and (2) not using high-power transmissions for a long time. The approach also assumed that the distance between all nodes in a cluster is equal in length, and for each step of WSN operation, a distinct fitness function was developed, improved, and tailored. The results demonstrated that the proposed method enhanced the average energy level by 13%, the coverage rate by 9.69% and the minimized overlap ratio by 35.43% compared with the existing approaches.

In [10] author introduces an Improved coverage and routing scheme using an Improved CSO procedure with Cauchy distribution coupled with a non-uniform clustering procedure which improves routing productivity. The coverage optimization achieves a maximum node coverage rate of 99.52% with minimal runtime, while the routing optimization scheme reduces energy loss to 0.84% and stabilizes in 1.47 minutes. Compared to traditional algorithms, the proposed CD-CSA and DCRUC algorithms offer higher coverage, faster convergence, and improved energy management. In [11] author introduces a Collaborative Harris Hawk Fuzzy Optimization (CHHFO), which combines fuzzy logic and HHO algorithms that work together to optimize energy consumption and extend the life of WSNs. For selection, it uses Mamdani’s fuzzy inference system, which reduces potential HHO hotspots to identify the best relay node for data transmission. This innovative adaptive function that considers power consumption and load balancing, simulation results show that CHHFO outperforms the existing protocol HHO-UCRA, providing higher network throughput. IHHO-F and EFCR are 8.76%, 11.73%, and 8.64%, respectively, and reduce energy consumption by 0.88%, 39.79%, and 34.25%. The CHHFO protocol shows good scalability and performance criteria. Future research can incorporate deep reinforcement learning to enhance the adaptability of WSNs in dynamic environments. The Hybrid Seagull and Whale Optimization Algorithm-based Dynamic Clustering Protocol (HSWOA-DCP) [12] designed to optimize CH selection, by minimizing unnecessary energy consumption. The proposed approach combines the exploration capabilities of the Seagull Optimization Algorithm (SEOA) with the exploitation strengths of the WOA by combining the spiral attacking principles of SEOA with the contraction mechanism of WOA, this hybrid approach effectively enhances the selection process and lowers the possibility of selecting fewer fit nodes, thereby addressing issues like premature convergence and computational accuracy during CH selection. Comparing HSWOA-DCP to current CH selection techniques, simulation results show that it greatly improves network survivability, residual energy, and overall throughput. Statistical analyses, including ANOVA tests, validate the proposed protocol’s energy stability across various data transmission rounds.

The hybrid K-means ant Lion Optimization method for Energy-efficient Routing (K-LionER) by using K-means for cluster formation and ALO for the best CH selection, the K-LionER scheme seeks to increase network lifespan and energy efficiency [13]. Metrics like remnant energy, the distance between CHs and BS, and intra-cluster communication cost are used to select the CHs, which collect data from their cluster members and relay it to the BS. K-LionER performs better than well-known protocols like LEACH, ECFU, and GADA-LEACH, according to a series of simulations carried out in MATLAB 2017a. Gains of 10% to 48% are achieved in alive node count, stability period, and overall network lifetime. [14] This work presents an Energy Efficient Routing with Cluster-based Genetic Algorithm (EER-CGA) designed to optimize energy consumption and routing especially in applications such as border surveillance and disaster management. The optimal data transmission channels are found using a genetic algorithm, so that they could minimize latency and maximize the lifetime of the network. Adaptive clustering depends on residual energy. The EER-CGA takes into account fitness functions related to energy, node centrality, and Euclidean distance with the aim of maximizing packet delivery ratios while minimizing energy expenditure. Compared to other methods such as LEACH-GA, CRCGA, and GAUCR, EER-CGA has shown better simulation results in terms of higher packet delivery ratios, energy consumptions, and reduced delays. The work proves efficiency of EER-CGA in terms of energy management of sensor nodes and optimization of data routes to enhance the lifetime of the network.

The authors of [15] presented an energy-efficient approach that selected the CH based on node density and used fuzzy-C means. The sensor nodes were organized into clusters utilizing the fuzzy C-means method. For communication within clusters, single-hop transmission was used, whereas multi-hop transmission was applied for communication between clusters. The proposed technique has been implemented in MATLAB, and the outcomes were compared in relation to the quantity of CHs, energy consumption, residual energy, and live nodes The author in [16] suggests a hybrid strategy to improve routing and clustering. This method combines two widely used metaheuristic strategies. The network is initially clustered using the Whale Optimization Algorithm (WOA), also referred to as WOA clustering, which aids in selecting the best CHs. Next, to facilitate communication between CHs and the BS, the HHO algorithm is employed for routing, or HHO-routing. It has been shown that implementing these tactics results in a considerable reduction of the energy required to transfer records to BS.

In [17], the authors introduced Binary Multi objective Fish Migration Optimization (BMAFMO) using a Pareto optimum solution method for boosting the global search and employed a sigmoid transformation function to pick the CH for each cluster in the network. The rank sum test was used to check the reliability of the proposed approach, and the study considered 8 test problems and 4 evaluation metrics. The simulation results were compared with those of several heuristic algorithms, such as HypE, IBEA, MOEAD, NSGA-II and MOEADUR.

In [18], the authors proposed the Quantum Beluga Whale Optimization (QBWO) algorithm, which centrally configures all the clusters within the network area, including the CH, cluster members, centroids, cluster priority and cluster validity period. The algorithm also cooperates with the subsequent switching stage and steady stage. When compared to other existing methods like LEACH, GA2-LEACH, and PE-LEACH using parameters like network load, network lifespan, and energy usage, the suggested QBSO algorithm performed better than the others. The authors of [19] suggested the Improved Fuzzy-C means (IFCM), which takes distance and energy into account as the primary factors. The best path was determined using Custom Honey Badger and Coot Optimization (CHBCO), which considers variables like energy and connection quality. Due to its fault tolerance efficiency, this method also manages CH failure. Members of the cluster may relocate to neighboring clusters if a CH fault is identified. When this strategy was contrasted with other strategies, the findings demonstrated that the suggested strategy beat all other strategies.

The authors of [20] addressed the issue of energy consumption while constructing clusters with sensor nodes and a CH by introducing an energy-efficient approach based on static clustering. The node with the most residual energy and the closest proximity to the most nodes in the cluster was named the CH. The proposed technique was implemented with MATLAB, and the results were compared with LEACH-DC, LEACH-SC, EEC, and EAC-ECHS in relation to energy utilization, node death rate, and longevity of network. Based on the criteria, the proposed method outperformed alternative strategies by 35%. To improve the global search capabilities, the authors of [21] introduced an energy-based clustering protocol that made use of the binary salp swarm method (ECBS). To increase energy efficiency, this study makes use of a gradient forwarding tree with two levels formed through a cost function. Network communication via single and multi-hop is made easier by routing. The approach was compared with strategies such as SEP, DDEEC, and BEECP using metrics including the number of dead nodes, packet delivery ratio, residual energy left in the network, network stability, and network life span. The proposed approach has shown significant improvement over other approaches in terms of the metrics considered. The authors of [22] suggested using the Ant Lion Optimization (ALO) method to combine nodes into clusters and the Geography-Enhanced Energy Efficient Routing (GEAR-R) technique to dynamically lower network overhead. The node coordinates are assumed to be static, or that the nodes’ locations are known, using the GEAR-R method. In terms of utilization of energy, latency, and lifespan of network, measures the results of this strategy were compared with those of the ACO, GHO, and MFO methods. To achieve load balancing with probability allocated to each node, the authors of [23] introduced the Fuzzy Balanced Cost CH Selection (FBECS), which predicts the eligibility index of nodes to be CHs. The technique was evaluated with BCSA and LEACH using network stability, longevity, load balancing and data forwarding parameters.

In [24], the authors proposed a zone-based energy-efficient clustering algorithm that aims to address scalability and sensitivity issues along with other metrics that could lead to energy efficiency, optimal routing, and network lifetime. The distance between the ZM and BS was considered a vital parameter because it determines the number of CHs required in the zone for nonuniform clustering. After choosing the number of CHs from the zone, the technique relies on the assumptions that cluster members can join any CH in the zone by using a fuzzy-based clustering approach, and cluster size is uniform at zone levels. The suggested method was contrasted in terms of usage of energy, packet reception and lifespan with the EEGBR, MFCL, and LEACH methods.

To solve the hot spot issue, reduce communication node distance, and choose the CH based on a fitness function incorporating crucial WSN factors, Genetic Algorithm Based Optimized Clustering (GAOC) is suggested in [25]. Multiple data sink-based GAOC was presented with performance indicators such as network lifespan, stability duration, dead nodes, throughput, and network remaining energy. This method was put into practice in MATLAB and compared with other approaches. The suggested method has significantly surpassed alternative methods in terms of overall performance. A Hybrid Artificial Bee Colony and Monarchy Butterfly-based Optimization algorithm (HABC-MBOA) is presented in [26] with the goal of preventing solutions from being trapped in local optima and of choosing the best CH throughout the clustering process. The inadequacy of ABC algorithm is eliminated to improve global search potential. This approach also aimed to avoid the CH overload; thus, rapid death of sensor nodes can be avoided. The results proved that HABC-MOBA performed 18.92% better than FFCGWO, FFOCR and HAS-PSO.

In [27], the author developed APCLO, a protocol that makes use of chaotic lion swarm optimization for greatest cluster formation. This method surpasses HSA, KH, and APSA in strength efficiency, throughput, and lengthening of the network lifespan, achieving upgrades of 5.9%, 15.3%, and 4.7%, respectively. To choose the best CH, a hybrid technique called Particle Distance Updated Sea Lion Optimization (PDU-SLno) is presented in [28]. The energy model is also established. i.e., whether it is a free space model or multipath model, based on distance from ‘permissible bit-error rate and node to the receiver’. In the event of re-clustering, the distance matrix establishes the threshold distance, and the Euclidian distance establishes the distance between the CH and other cluster member nodes.

In [29], the authors included a dual CH coupled with an interval-based method for CH re-election. The aim of this strategy is to reduce the energy expenditure related to frequent cluster formation. A complex hybrid swarm intelligence optimization set of rules known as GWOA-CH is produced because of considering residual power and distance to BS over the duration of CH election. This approach combines the Whale Optimization approach (WOA) with Gray Wolf Optimization (GWO) to improve the effectiveness of CH election. The author of [30] put forth a dependable, energy-conscious cluster-based multi-hop routing method that permits cluster reorganization as necessary while routing takes place. By distributing energy throughout the network,it is also guaranteed to use less energy than existing multi-hop routing methods. This approach combines the Genetic Algorithm with the K-means and Open-Source Development Model Algorithm (ODMA) based clustering techniques to perform multi-hop routing. The DPFCP (Distributed Particle Swarm Optimization-Based Fuzzy Clustering Protocol) is suggested by the authors in [31] as an effective way to control steady power consumption and strength intake in WSNs. This protocol chooses CHs mostly based on node degree and residual strength metrics using a Mamdani fuzzy inference method. Furthermore, the basic rules for CH choosing are improved with the use of a PSO technique. Non-CH nodes belong to clusters mostly because of factors like final strength, which increases power efficiency, and how close the node is to the CH. DPFCP furthermore contains an on-demand approach to retain clusters both locally and globally, consequently minimizing computational and communication overheads to preserve energy.

The study described in [32] presents the Unequal Clustering and Routing Algorithms (CSO-UCRA) based on competitive Swarm Optimization. By considering variables including sensor dispersion, node distances, and energy levels, this approach effectively chooses CHs. This method saves a significant amount of energy and increases the network’s lifetime by 56.92% without having GPS on every CH sensor node. In [33], researchers advanced an underwater clustering protocol known as FCMMFO to enhance the performance of Underwater WSN (UWSNs). This protocol integrates the fuzzy c-method (FCM) for determining the optimal range of clusters and moth-flame optimization (MFO) for selecting suitable CH nodes. However, FCMMFO lacks a multi-hop mechanism that can further enhance network performance.

The authors of [34] introduced the Hybrid Optimized Energy Efficient Adaptive Clustered Routing (HOEEACR) approach, which combines the African Vulture Optimization (AAVO) algorithm, the Aquila algorithm, and the Genetic Bee Colony (GBC) algorithm. The GBC and AAVO algorithm select CHs and optimal routing primarily based on standards inclusive of proximity to neighboring nodes, residual strength, node levels, and centrality respectively. To further enhance the CH selection process, the authors of [35] have proposed improvements. Suggested a clustering-based routing method that combines the Levy mutation with the Sine Cosine method. This method adjusts the number of CHs according to the availability of nodes and selects highly capable nodes as candidates for CH selection. The algorithm includes a health feature that considers intra-cluster distances to create a certain uniform cluster distribution. The CH election technique employs an enhanced Sine Cosine Algorithm with an optimized step length seek and Levy mutation to maintain populace dynamics.

The authors of [36] presented the Energy Efficient Ultra-Scalable Ensemble Clustering (EEU-SEC) technique, which can handle big datasets, for effectively clustering nodes in WSNs. The Flamingo Search Algorithm (FSA) is chosen for CH selection because it offers both stability and low computational complexity. Q-Learning is then applied to improve performance by identifying the most efficient path between the CHs and the BS by factoring in coverage, distance, and energy consumption, this method enhances overall network performance and extends its lifespan.

Table 1 shows the overall summary of existing approaches. Literature review indicates that while both optimization-based and clustering-based approaches are effective at addressing network challenges, each has its own set of limitations. Optimization-based strategies offer precise control over network resources, but they are often complex to implement. Conversely, clustering-based approaches depend heavily on specific network conditions to perform optimally. The EECR-GeMACHHOA approach offers several distinctive benefits that distinguish it from current methods by integrating parameters such as central point, node connectivity, communication range, node suitability score, and Euclidean distance for ZM selection, this approach provides a comprehensive strategy that has not been used in previous algorithms. This multifaceted approach significantly enhances its effectiveness and applicability. Moreover, EECR-GeMACHHOA prioritizes energy-efficient CH selection through a hybrid strategy that combines modified ACO algorithm using dynamic pheromone evaporation with the enhanced HHO technique. This approach enables optimal cluster formation and incorporates an energy-conscious multi-hop routing strategy, optimizing both energy usage and overall network performance by resolving the hotspot problem via optimal routing path selection, this algorithm achieves a more even distribution of energy across the network, thereby extending its operational lifespan. Advanced integration of clustering and routing techniques in EECR-GeMACHHOA effectively overcomes these limitations. This offers a balanced and all-encompassing solution for WSNs by extending the network lifetime, lowering energy consumption, and improving overall network performance.

**Table 1:** Summary for CH Detection and Routing.

Method	CH & Routing Selection	Key Features	Obstacles	Future Scope
APCLO [27]	Chaotic Lion Swarm, Multi-hop routing	Enhances clustering, improves energy	Scalability issues	Dynamic environmental adaptation
GWOA-CH [29]	Fitness Function, Multi-hop routing	Reduces energy, dual CHs	Overhead in dual CHs	Adaptation to mobility
DPFCP [31]	Fuzzy Inference, PSO, Single/Multi-hop	Minimizes energy use, cluster maintenance	Complexity with fuzzy	Lightweight real-time optimization
HOEE-ACR [34]	AAVO, GBC, Aquila Optimization, Adaptive routing	Combines optimization algorithms, energy	Compu-tational overhead	Simplified model for scalability
EEU-SEC [36]	Flamingo Search, Q-Learning, Multi-hop routing	Handles large datasets, optimal paths	Dynamic environment complexity	AI-based decision-making
PDU-SLno [28]	Distance-based, PSO, Multi-hop routing	Euclidean distance for optimization	Conver-gence in dense networks	Convergence improvement
GAOC [30]	Genetic Algorithm, Multi-hop routing	Combines GA, K-means, ODMA	High complexity	GA parameter optimization
CSO-UCRA [32]	Competitive Swarm Optimization, Unequal Cluster Routing	Improves energy efficiency	Static node dependence	Mobile WSN applications

## Proposed work

In this section, an improved hybrid HHO algorithm is presented, which finds the best multi-hop paths between ZM, CHs, and BS. The modified ACO algorithm with dynamic pheromone evaporation is proposed for multiple CH selection, and a novel geometric translational symmetry feature is applied for ZM selection which reduces energy consumption and raising packet delivery ratio, these ideal pathways boost network performance and extend network lifetime.

### Network model

**Fig 2:**
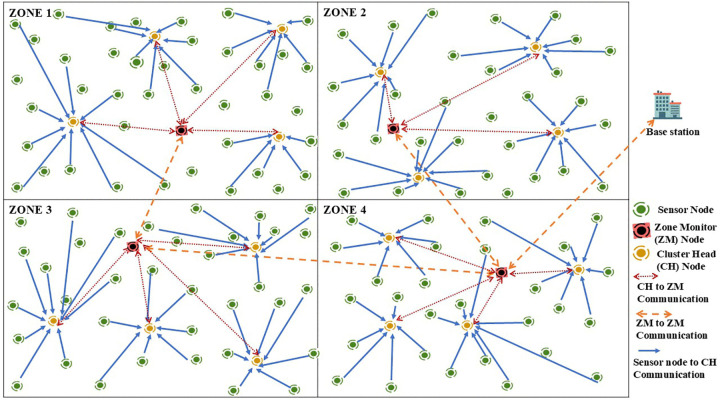
Proposed EECR-GeMACHHOA Network architecture.

The random deployment model for border surveillance within a WSN aims to demonstrate its impact on network efficiency and longevity. The proposed network architecture, as illustrated in Fig 2, features numerous sensor nodes with limited resources and one BS with ample resources, with symmetric wireless links between nodes. The area under surveillance is managed through random placement of sensors with the following assumptions:

1. A specific number of sensors are randomly distributed across the surveillance area.
2. Deployment and surveillance activities occur within a flat, two-dimensional environment.
3. Sink nodes are fixed at designated locations, serving as central points for data collection and communication.
4. The deployment area is segmented into zones, each with one ZM responsible for managing communication with the sink node and coordinating multiple CH within the zone.
5. All sensors have identical sensing ranges, transmission ranges, and energy characteristics to ensure consistent performance.
6. Sensors and ZMs adjust their communication strategies based on their positions and the network’s topology.
7. Sensors detect intruders and relay the data to the ZM, where it is aggregated and then forwarded to BS.
8. Efficient utilization of sensor nodes is essential to prevent energy imbalances and maintain optimal network operation.
9. The random deployment model allows for tracking intruder trajectories based on sensor data, with effectiveness dependent on sensor placement.
10. The random sensor placement and the effectiveness of the data gathering and transmission operations affect the network lifetime.

**Fig 3:**
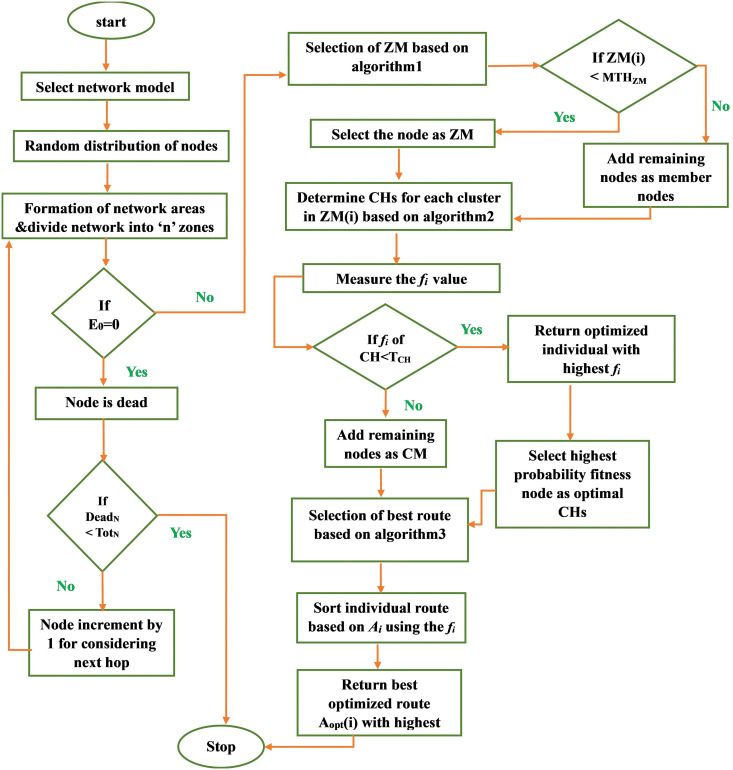
Diagramatic flow of proposed EECR-GeMACHHOA approach.

Figure 3 provides a diagrammatic flow of the proposed approach, detailing how data is processed and communicated from sensor nodes through CHs and ZMs to the sink node. This deployment model supports dynamic coverage and adaptability, critical for effective border surveillance.

#### Consumption of energy model.

The main problem in WSNs is making use of the sensor nodes’ limited battery power. Nodes are incapable of providing energy as battery energy drops because they lack re-energizing characteristics. A node’s battery power may be used for sensing, hearing, receiving, aggregating, and transmitting. The node is capable of transmitting X bytes of data over a distance d to the receiver, utilizing either the free space model $E_{rs}\cdot d^{2}$, or the multipath model $E_{pw}\cdot d^{4}$, depending on the distance and the energy consumption $E_{cs}$ required for data aggregation. This process is influenced by the node’s electronic energy, $E_{el}$ and its proximity to the allowable bit-error rate. This study uses the same energy model, meaning that amplification is not considered. Equation 1 represents the overall energy used for the complete data transmission operation.


$$\begin{array}{c} ER_{tr}(X,d)=\left\{ \begin{array}{cc} X\cdot (E_{el}+E_{rs}\cdot d^{2}), & \text{when }d<d_{0}\\ X\cdot (E_{el}+E_{pw}\cdot d^{4}), & \text{when }d\geq d_{0} \end{array}\right. \end{array}$$ (1)

The total energy $ER_{tr}$ required to transmit X bytes of data over a distance d, along with the energy $E_{cs}$ used for data aggregation, is equal to the electronic energy $E_{el}$ consumed by the nodes for transmission. Likewise, equation 2 represents the energy needed to receive M bytes of data across a distance d.


$$\begin{array}{c} ER_{rv}(X,d)=E_{el}\cdot X \end{array}$$ (2)

In equation 3, the distance *d*_0_ is calculated using the amplifier energy $ER_{am}$ and the energy required while employing the free space energy model $E_{fm}$.


$$\begin{array}{c} d_{0}=\sqrt{\frac{E_{fm}}{ER_{am}}} \end{array}$$ (3)

Therefore, the total network energy reduction during the entire transmission process from a node to the receiver is given in equation 4,


$$\begin{array}{c} E_{tot}=ER_{tr}+ER_{rv}+E_{id}+E_{ct} \end{array}$$ (4)

were $E_{id}$ is the energy spent by a node while idle and $E_{ct}$ is the energy consumed by nodes for overall transmission or sensing.

#### Zone monitor selection.

In WSNs, effectively managing the limited resources of sensor nodes, particularly their battery energy, is essential for extending the network lifespan. ZM is selected using Algorithm 1, the selection option is crucial for optimizing energy usage and enhancing network efficiency by purposefully assigning particular nodes to recognize and control activities inside discrete zones. Numerous input parameters are used by the set of rules: the whole set of zones N, the coordinates of each node $\left(x_{i},y_{i}\right)$, the node connectivity information “Adjacency Matrix”$a_{ij}$, and each node’s residual power $Res_{pow}[j]$. In addition, a scaling element α=0.5 is applied in the lowest threshold price calculation $MTH_{ZM}$ to guarantee a fair distribution of energy across the various selected zone coordinators *ZM*(*i*) for each zone *Z*(*i*). The ZM’s duty is to oversee and coordinate the actions inside its specific zone to contribute to an extra energy-efficient and strong network operation.

**1. Calculation of the Geometric Center**

Each zone *Z*(*i*) is characterized by a set of nodes with specific coordinates $\left(x_{i},y_{i}\right)$. The algorithm first computes the geometric center $\left(X_{c},Y_{c}\right)$ of each zone to determine the central point around which node activities will be organized. This is essential for optimizing communication distances and minimizing energy consumption.


$$\begin{array}{c} X_{c}=\frac{\sum_{k\in Z(i)}x_{k}}{\text{count of nodes in }Z(i)} \end{array}$$ (5)

$$\begin{array}{c} Y_{c}=\frac{\sum_{k\in Z(i)}y_{k}}{\text{count of nodes in }Z(i)} \end{array}$$ (6)

**2. Calculate the Distance to the Center of Each Node**

To evaluate node proximity to the zone’s center, the algorithm computes the Euclidean distance $d_{j}$ for each node *j* within zone *Z*(*i*). The Euclidean distance metric, defined in the equation, measures the straight-line distance between each node and the geometric center. This distance metric is critical because it directly influences the energy required for communication and facilitates the selection of nodes that are centrally located and thus more energy-efficient for monitoring tasks.


$$\begin{array}{c} d_{j}=\sqrt{(x_{j}-X_{c}{)}^{2}+(y_{j}-Y_{c}{)}^{2}} \end{array}$$ (7)

**3. Calculate the Connectivity for Each Node**

Connectivity $C_{j}$ of node *j* in zone *Z*(*i*) is determined by summing the adjacency matrix values $a_{jk}$. This metric reflects the node’s ability to communicate effectively with other nodes within its zone. Nodes with higher connectivity values are preferred because they facilitate robust data transmission and network reliability.


$$\begin{array}{c} C_{j}=\sum_{k\in Z(i)}a_{jk} \end{array}$$ (8)

**4. Node Score Selection**

The algorithm computes a score $S_{j}$ for each node *j* in zone *Z*(*i*), integrating its residual energy, distance to the $Res_{pow}[j]$ center $d_{j}$, and connectivity $C_{j}$. Here, ϵ is a small constant introduced to handle cases where $d_{j}$ approaches zero, ensuring that the algorithm remains stable and avoids division by very small numbers. This scoring mechanism prioritizes nodes that possess sufficient energy reserves, are close to the zone center, and exhibit strong connectivity, thereby enhancing their suitability as ZM.


$$\begin{array}{c} S_{j}={\text{Res}}_{\text{pow}}[j]\cdot \left(\frac{1}{d_{j}+\epsilon }\right)\cdot C_{j} \end{array}$$ (9)

**5. Assigning Zone Monitor** The *ZM*(*i*) is determined by taking the node *j* with the greatest score $S_{j}$


$$\begin{array}{c} \text{ZM}(i)=\text{arg}\max_{j\in Z(i)}S_{j} \end{array}$$ (10)

This strategic selection ensures that each zone is overseen by a node capable of efficiently managing its resources and coordinating communications, thereby optimizing network performance and longevity.

**6. Determination of the Minimum threshold Value of Zone Monitors**

The algorithm calculates the minimum threshold value $MTH_{zm}$ to coordinate and potentially reassign *ZM* if their energy levels fall below acceptable limits:


$$\begin{array}{c} {\text{MTH}}_{\text{zm}}=\alpha \cdot \left(\frac{1}{N}\sum_{j=1}^{N}{\text{Res}}_{\text{pow}}[j]\right) \end{array}$$ (11)

By scaling the average residual energy across all nodes by α, the algorithm sets a baseline energy threshold that helps maintain network stability and ensures that ZM can effectively perform their duties over extended periods.

**7. Monitor and Re-Clustering**

Continuous monitoring of ZM ensures proactive management of node energy levels. To preserve the ideal energy distribution, reassignment is started when the remaining energy of any zone coordinator *ZM*(*i*) drops below the minimal threshold value $MTH_{zm}$. The *ZM*(*i*) is reassigned to the node with the greatest residual energy inside each zone after iteratively inspecting all zones *Z*(*i*). This process is mathematically expressed as:


$$\begin{array}{c} \sum_{i=1}^{N}\left({\text{Res}}_{\text{pow}}[\text{ZM}(i)]<{\text{MTH}}_{\text{ZM}}\right) \end{array}$$ (12)

$$\begin{array}{c} \text{ZM}(i)=\text{arg}\max_{j\in Z(i)}{\text{Res}}_{\text{pow}}[j] \end{array}$$ (13)

Equations 12, 13 contribute to the network’s lifespan extension by guaranteeing that the node with the most leftover energy in each zone is chosen as the ZM. The ZM is chosen to balance the energy load across the network and extend its overall lifetime by considering each zone’s greatest residual energy.

**Algorithm 1 Zone monitor selection and clustering**

1: **Input Parameters**

2: N←Total number of zones

3: Res_pow[j]←Residual energy of node j

4: $(x_{i},y_{i})\leftarrow \text{Coordinates of node }i$

5: $\text{Adjacency Matrix}[a_{ij}]\leftarrow \text{Connectivity information between nodes }i\text{ and }j$

6: α←0.5 ▷ Scaling factor for threshold

7: **Output:** Zone coordinator for each zone *ZC*(*i*)

8: **for** i←1 **to** *N* **do**

9:  **Calculate Geometric Center** based on equations 5 and 6

10:  **for** each node *j* in *Z*(*i*) **do**

11:   **Calculate Distance to center for each node** based on 7

12:   **Calculate Connectivity for each node** based on 8

13:  **end for**

14:  **Assign Zone Monitor** based on highest residual energy:

15:  ZC(i)←argmax(Res_pow[j]) for all *j* in *ZC*(*i*)

16:  **Determine Minimum Threshold Value for Zone Monitor** based on 11

17:  **Monitor Residual Energy and Re-cluster if Necessary**

18:  **while** ∑(Res_pow[ZC(i)])<MTHZC **do**

19:   **for** i←1 **to** *N* **do**

20:    **if** Res_pow[ZC(i)]<MTHZC **then**

21:     **Calculate** based on 12

22:    **end if**

23:   **end for**

24:  **end while**

25: **end for**

#### Modified ant colony with dynamic pheromone evaporation for multiple cluster head selection.

When a WSN is organized into multiple zones, with each zone containing several clusters and each cluster having its own CH, it becomes crucial to determine the expected number of CHs. This is especially important as the size of the zones varies. In Algorithm 2, the modified ant colony with dynamic pheromone evaporation method for multiple CH selection is described, considering important parameters like residual energy, node degree, distance, and energy efficiency. A population of ants is deployed, where each ant explores potential CH candidates within its designated zone. Each ant constructs a solution by probabilistically selecting nodes to become CHs. The main objective of having more CH within the zone is to establish an energy-efficient network with high reachability while prolonging the network and node lifetime. The potential CH candidates within each zone were identified. These nodes should have the capability to perform data aggregation and communication tasks. The assumptions are as follows:

1. *N* nodes randomly spaced out throughout the cluster.

2. The density of clusters is constant since the nodes are stationary, and the node density ρ is represented as:


$$\begin{array}{c} \rho =\frac{N}{\text{Transmission Area (A)}} \end{array}$$ (14)

3. The sensor node *N*_tr_ transmission range is calculated as:


$$\begin{array}{c} N_{\text{tr}}=\sqrt{\frac{\ln\left(\frac{1}{1-P_{\text{non-iso}}^{\frac{1}{n}}}\right)}{\rho \pi }} \end{array}$$ (15)

4. $P_{\text{non-iso}}$ determines the probability of the threshold that defines the non-isolation of the nodes in the given zone. The natural logarithm ln is used to transform the probability.

5. For each *N* in its transmission range, find neighbors *ne*. An indicator function *I* = 1 when the condition is met and 0 when it is not. Let $n_{d}$ denote the node degree:


$$\begin{array}{c} n_{d}=\sum_{ne\in N(d)}\frac{1}{\text{Dist}(d,ne)}\cdot I\left(\text{Dist}(d,ne)\leq N_{\text{tr}}\right) \end{array}$$ (16)

6. For each node within the cluster, calculate the degree difference, where $D_{di}$ determines the node degree of the current position and γ determines the predefined or expected node degree:


$$\begin{array}{c} D_{di}=d(n_{d},\gamma ) \end{array}$$ (17)

7. Find the average distance of member nodes by computing the sum of distances, where $ne_{i}$ denotes the *i*th neighbor of node *d* and $tn_{d}$ represents the total number of neighbors:


$$\begin{array}{c} {\text{Dist}}_{\text{sum}}=\sum_{i=1}^{tn_{d}}\text{Dist}(d,ne_{i}) \end{array}$$ (18)

$$\begin{array}{c} {\text{Dist}}_{\text{avg}}=\frac{1}{tn_{d}}\sum_{ne\in N(d)}\text{Dist}(d,ne) \end{array}$$ (19)

8. Finding the node with the highest residual power, where RES_en_(*d*) is the residual energy with respect to node *d*, and ${\text{RES}}_{i}(d)$ is the initial energy with respect to node *d*:


$$\begin{array}{c} {\text{RES}}_{\text{ht}}=\frac{{\text{RES}}_{\mathrm{en}}(d)}{{\text{RES}}_{i}(d)} \end{array}$$ (20)

1. **probablistic cluster head selection** The pheromone value $\tau_{i}$ and heuristic information $\eta_{i}$ of a node *i* determine its local desirability based on the node properties likelihood $p_{i}$ of being chosen as a CH by an ant:


$$\begin{array}{c} P_{i}=\frac{(\tau_{i}{)}^{\alpha }\cdot (\eta_{i}{)}^{\beta }}{\sum_{j\in N}(\tau_{j}{)}^{\alpha }\cdot (\eta_{j}{)}^{\beta }} \end{array}$$ (21)

where the pheromone and heuristic values are controlled by α and β.

2. **Dynamic Pheromone Evaporation Rate** The pheromone evaporation rate ρ(t) is adjusted by the Dynamic Pheromone Evaporation Rate in accordance with the node’s average residual energy


$$\begin{array}{c} \rho (t)=\rho_{\text{min}}+(\rho_{\text{max}}-\rho_{\text{min}})\cdot \left(1-\frac{{\text{RES}}_{\text{avg}}(t)}{{\text{RES}}_{\text{init}}}\right) \end{array}$$ (22)

An ant’s pheromone deposit on node *i* is determined by the solution’s quality (fitness value)


$$\begin{array}{c} \Delta \tau_{i}=\frac{Q}{f_{\text{best}}} \end{array}$$ (23)

where *Q* is a constant representing the pheromone strength, and *f*_best_ is the best solution’s fitness value found during the ant’s exploration. After each iteration, ants deposit pheromone on the nodes they visit. The pheromone update equation is


$$\begin{array}{c} \tau_{i}=(1-\rho (t))\cdot \tau_{i}+\Delta \tau_{i} \end{array}$$ (24)

The amount of pheromone deposited on node *i* is $\Delta \tau_{i}$, and it is proportional to the quality of the solution. A fitness function is designed to evaluate each possible node based on RES_ht_, *N*_tr_, $n_{d}$, distance to ZM, and $D_{di}$. Each node is given a score by the fitness function, which considers these factors. The fitness function $f_{i}$ for node *i* within a zone is now expressed as:


$$\begin{array}{c} f_{i}=w_{1}\cdot \left(\frac{1}{E_{\text{total}}}\right)+w_{2}\cdot \left(\frac{1}{D_{di}}\right)+w_{3}\cdot \left(\frac{1}{{\text{Dist}}_{\text{avg}}}\right)+w_{4}\cdot {\text{RES}}_{\text{ht}} \end{array}$$ (25)

where *E*_total_ represents the total energy consumption, $D_{di}$ represents the degree difference, and *w*_1_, *w*_2_, *w*_3_, *w*_4_ are the weighting factors that sum to 1.

3. **Cluster Head Selection and Verification** After all ants have completed their exploration, the nodes with the highest pheromone values within each zone are selected as CHs.Pheromone Value Ranking for each zone $Z_{j}$, nodes are ranked based on their pheromone values $\tau_{i}$:


$$\begin{array}{c} {\text{Rank}}_{i}=\text{Sort}(\tau_{i})\;\text{for }i\in Z_{j} \end{array}$$ (26)

$$\begin{array}{c} i_{\text{suboptimal}}=\text{arg}\min_{i\in {\text{CH}}_{j}}f_{i} \end{array}$$ (27)

where Sort is a function that orders nodes in descending order of their pheromone values. If the fitness value $f_{i}$ for a selected CH falls below a predefined threshold *T*_CH_, it is replaced with a node from the same zone that has the highest residual energy RES_ht_. Identify the suboptimal CH (*i*_suboptimal_) with the lowest $f_{i}$ in the set of current CHs within zone $Z_{j}$. Once the suboptimal CH is identified, replace it with the node that has the highest residual energy RES_ht_ from the replacement candidates. Once the new CHs are selected, a message is broadcast to all nodes to inform them of the updated CH assignments.


$$\begin{array}{c} i_{\text{new}}=\text{arg}\max_{i\in {\text{Replacement}}_{j}}{\text{RES}}_{\text{ht}}(i) \end{array}$$ (28)

$$\begin{array}{c} {\text{CH}}_{j}^{\text{new}}=({\text{CH}}_{j}⧵\{i_{\text{suboptimal}}\})\cup \{i_{\text{new}}\} \end{array}$$ (29)

**Algorithm 2 Modified ant colony algorithm with dynamic pheromone evaporation for multiple cluster head selection**

1: **BEGIN**

2: **Input:** *N* (number of nodes), *Z* (number of zones)

3: Divide network into *Z* zones

4: **for** each node *i* in *N* **do** ▷ Initialize pheromone levels

5:  $\tau_{i}\leftarrow \tau_{\text{initial}}$

6: **end for**

7: **for** each zone *z* in *Z* **do**

8:  Deploy *M* ants

9: **end for**

10: **for** each ant *k* in *M* **do** ▷ Ant-based CH selection

11:  **for** each node *i* in zone *z* **do**

12:   $n_{d},D_{di}\leftarrow$ Equation 16, 17

13:   ${\text{Dist}}_{\text{avg}}(i)\leftarrow$ Equation 19

14:   ${\text{RES}}_{i}\leftarrow$ Equation 20

15:  **end for**

16:  **for** each node *i* in zone *z* **do**

17:   $P_{i}\leftarrow$ Equation 21

18:  **end for**

19:  Select node with $\max P_{i}$ in zone *z* as CH

20: **end for**

21: ρ(t)← Equation 22 ▷ Update pheromone values

22: **for** each node *i* in *N* **do**

23:  $\Delta \tau_{i}\leftarrow$ Equation 23

24:  $\tau_{i}\leftarrow$ Equation 24

25: **end for**

26: **for** each selected CH node *i* in zone *z* **do** ▷ Evaluate fitness of CH candidates

27:  $f_{i}\leftarrow$ Equation 25

28: **end for**

29: **for** each selected CH node *i* in zone *z* **do** ▷ Replace suboptimal CHs

30:  **if** $f_{i}<T_{\text{CH}}$ **then**

31:   $i_{\text{suboptimal}}\leftarrow$ Equation 27

32:   $i_{\text{new}}\leftarrow$ Equation 28

33:   ${\text{CH}}_{j}^{\text{new}}\leftarrow$ Equation 29

34:  **end if**

35: **end for**

36: **END**

#### Multihop communication and optimal routing for EECR-GeMACHHOA algorithm.

The EECR-GEMACHHOA algorithm offers a cutting-edge method which combines clustering mechanisms with metaheuristic optimization techniques. Algorithm 3 shows the optimal cluster-based routing for EECR-GeMACHHOA algorithm. This algorithm brings together geometric translational symmetry, dynamic pheromone evaporation, and HHO to boost energy efficiency, scalability, and flexibility in changing network conditions.

EECR-GeMACHHOA shines in picking the best ZM and CHs and setting up effective routing paths while keeping energy spread across the network. Its flexible zone-based clustering system makes sure energy use is uniform, with regular updates of ZM and CHs to make the network last longer. A modified ACO helps choose the right CHs by looking at several factors like how well nodes connect, leftover energy how far they can send data, and average distance this ensures data gets gathered and sent within zones. If the network gets disrupted EECR-GeMACHHOA quickly finds other routes using the search and use phases of HHOA to bounce back fast. Direct transmission and multi-hop transmission are the two main methods used to transfer packets from ZMs to BSs.

Direct communication includes the ZM transmitting data immediately to the BS after gathering and processing information from its member nodes. While this approach is energy-efficient for ZMs close to the BS, it could be more energy consuming for those farther away. Additionally, network growth is limited by the sensors transmission range, which limits it to the greatest distance at which the farthest ZM can instantly connect with the BS. This method is most effective when the transmission distances are short. Alternatively, multi-hop transmission involves relaying packets through multiple ZM nodes before they reach the BS. This method is particularly effective for sensor networks with many closely spaced nodes. Multi-hop communication boosts energy efficiency by avoiding high-power transmissions, mitigating signal propagation issues over long distances, and enhancing the network’s lifespan and scalability. Multi-hop transmission typically results in better load distribution and a longer network lifespan than direct communication, particularly in larger and extra dispersed WSN networks. Data is exchanged between nodes and CHs and from the CH to ZM. During the exploration stage, it identifies the possible routes, to redirect data to the BS. Every hawk’s position is updated based on best answer found so far (prey), exploration of search space, and random tactics. This technique additionally eliminates distance-associated constraints and keeps less data transmission delay by making the network stronger and more reliable. Its ability to modify dynamically to handle shifts in node density and give the optimum routing options. This allows for rapid, energy-saving communication between ZH and the BS.

1. **Exploration Phase** The random numbers *r*, *r*_1_, *r*_2_, *r*_3_, and *r*_4_ are uniformly distributed in the range [0, 1]. The positions of the hawk at iteration *t* are indicated by *A*(*t*), the prey’s (best solution found so far) position is shown by $A_{p}(t)$, and the randomly selected position from the current population is shown by *A*_rand_(*t*). *LB* and *UB* stand for the search space’s lower and upper boundaries, respectively. ℋ(x) is *t*he Heaviside step function, which returns 1 when x≥0 and 0 otherwise. The position update formula during the exploration phase, based on the current population, best solution, and search bounds, is given by:


$$\begin{array}{cc} A_{\text{zm}}(t+1)= & \;A_{\text{rand}}(t)-r_{1}\cdot \left|A_{\text{rand}}(t)-2r_{2}\cdot A(t)\right|\cdot ℋ(r-0.5)\\ & +\left(A_{p}(t)-A(t)-r_{3}\cdot \left(LB+r_{4}\cdot (UB-LB)\right)\right)\cdot ℋ(0.5-r) \end{array}$$ (30)

2. **Exploitation Phase** Hawks were concerned about the effectiveness of the paths they discovered during exploration, as they converged toward the prey at some point during the exploitation phase. During this period, several strategies are employed, including progressive brief dives, hard besiege, and mild besiege.*E* denote the energy of the prey, which decreases over iterations. *J*(*t*) represent the jump strength of the hawk at iteration *t*. ΔA(t) indicate the difference between the positions of the prey and the hawk at iteration *t*.t−(u,y) represent random numbers with a uniform distribu*t*ion in the range [0, 1].


$$\begin{array}{c} A_{\text{zm}}(t+1)=\left\{ \begin{array}{cc} A_{p}(t)-E\cdot |J(t)\cdot A_{p}(t)-A(t)|, & \text{if }|E|\geq 0.5,\\ \Delta A(t)\cdot e^{r}\cdot \cos(2\pi r), & \text{if }|E|<0.5, \end{array}\right. \end{array}$$ (31)

3. **Mild Besiege** The hawk moves up in the soft besiege method by converting the distance between its current position and the prey’s position. The hawk’s next position, *A*(*t* + 1), is determined based on the calculated difference ΔA(t), which represents the difference between the prey and the hawk positions at iteration *t*. This method allows the hawk to slowly besiege *t*he prey by maintaining a calculated distance, simulating a gentle approach to catching the prey.


$$\begin{array}{c} A(t+1)=\Delta A(t)-E\cdot |J(t)\cdot A_{p}(t)-A(t)| \end{array}$$ (32)

$$\begin{array}{c} J(t)=2(1-u) \end{array}$$ (33)

$$\begin{array}{c} \Delta A(t)=A_{p}(t)-A(t) \end{array}$$ (34)

4. **Hard Besiege** In the hard besiege method, the hawk aggressively closes in on the prey by updating its position *A*(*t* + 1). The update is achieved by subtracting the product of the prey’s energy and the gap to the prey from the prey’s position. This approach simulates a more aggressive method, where the hawk rapidly decreases the distance in each iteration.


$$\begin{array}{c} A(t+1)=A_{p}(t)-E\cdot |\Delta A(t)| \end{array}$$ (35)

5. **Progressive Rapid Dives** This strategy simulates a hawk making rapid, aggressive dives toward the prey. The hawk’s movements are influenced by distance and jump strength, resulting in dynamic and varied approaches. The inclusion of exponential and periodic components makes the hawk’s movements less predictable to the prey. The position update formula is given by:


$$\begin{array}{c} A(t+1)=\left\{ \begin{array}{cc} A_{p}(t)+\Delta A(t)\cdot e^{y}\cdot \cos(2\pi y), & \text{if }|J(t)|\geq 1,\\ |J(t)\cdot X_{p}(t)-A(t)|, & \text{if }|J(t)|<1, \end{array}\right. \end{array}$$ (36)

6. **Optimal Routing path Selection** The optimized routing path *A*_opt_(*i*) is selected by evaluating potential paths using a fitness function. The path with highest fitness value is chosen for data transmission. The selection aims to maximize network lifetime and minimize energy consumption while avoiding unreliable nodes. Mathematically, the optimized path is selected as:


$$\begin{array}{c} A_{\text{opt}}(i)=\text{arg}\max_{A(i)\in 𝒫}\text{f}(i) \end{array}$$ (37)

𝒫 is the set of all possible routing paths for node i,*A*(*i*) represents a specific routing path for node i, *f*(*i*) is the fitness value of the routing path *A*(*i*), argmax identifies the routing path *A*(*i*) with the maximum fitness value.

**Algorithm 3 Hybrid zone-based clustering and optimal routing for EECR-GeMACHHOA algorithm**

1: **BEGIN**

2: **Input:** $N_{z}$ (Total zones), *ZM*(*i*) (ZM for each zone *i* based on Algorithm 1), *CHs* (Selected Cluster Heads per zone based on Algorithm 2)

3: **Output:** Optimized routing paths from *ZM* to *BS*

4: **for** each zone *Z*(*i*) in $N_{z}$ **do**

5:  Initialize *ZM*(*i*) using Algorithm 1

6:  Initialize *CHs* using Algorithm 2

7: **end for**

8: **for** each *ZM*(*i*) in $N_{z}$ **do**

9:  Initialize position *A*(*i*) of routing path from *ZM*(*i*) to *BS* using HHO

10:  **repeat** ▷ Until convergence criteria are met

11:   **if** in Exploration Phase **then**

12:    Select random position *A*_rand_(*t*) from current *A*(*i*)

13:    Generate random numbers $r_{1},r_{2},r_{3},r_{4}$

14:    **if** r≥0.5 **then**

15:     Update position:

$A(i)(t+1)\leftarrow A_{\text{rand}}(t)-r_{1}\cdot |A_{\text{rand}}(t)-2\cdot r_{2}\cdot A(i)(t)|$

16:    **else**

17:     Update position:

$A(i)(t+1)\leftarrow (A_{p}(t)-A(i)(t))-r_{3}\cdot (LB_{\text{Routing}}+r_{4}\cdot (UB_{\text{Routing}}-LB_{\text{Routing}}))$

18:    **end if**

19:   **end if**

20:   Evaluate fitness function *F*(*i*) for the routing path *A*(*i*) based on Equation 25

21:   **if** in Exploitation Phase **then**

22:    Calculate prey position based on Equations 31, 32

23:    Generate random numbers *u*, *y*

24:    Calculate jump strength $J_{A}(i)(t)$

25:    **if** $|J_{A}(i)(t)|\geq 1$ **then**

26:     Update position based on Equation 35

27:    **else**

28:     Update position based on Equation 36

29:    **end if**

30:   **end if**

31:  **until** convergence criteria are met

32: **end for**

33: **for** each *ZM*(*i*) in $N_{z}$ **do**

34:  Select the optimized routing path *A*_opt_(*i*) based on equation 37

35: **end for**

36: **END**

## Simulation setup and evaluation metrics

**Fig 4:**
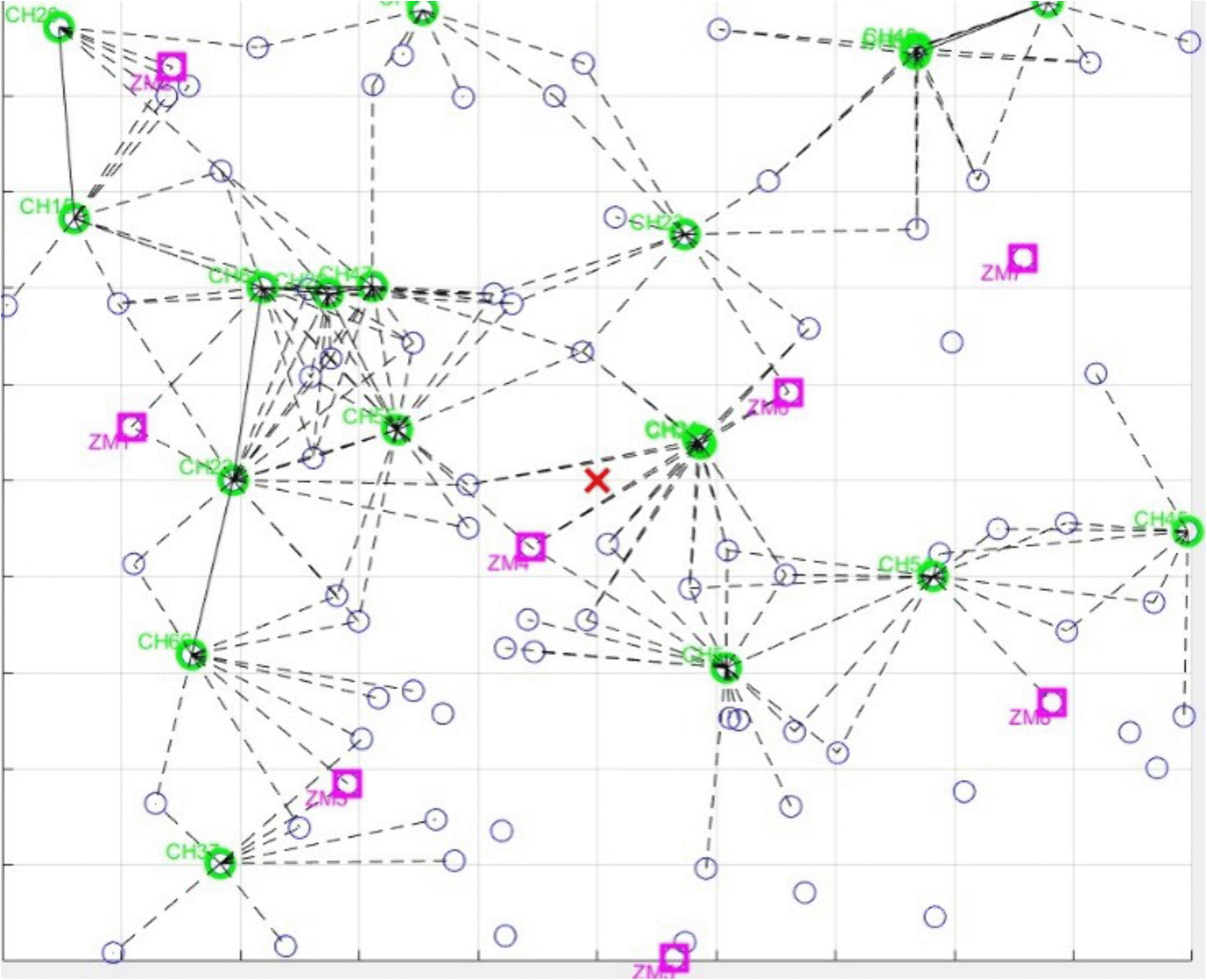
Structure of zone based clustering sensor network.

This part of the paper discusses the experimental setup, hardware configurations, simulation parameters and setup values used to construct the network in the MATLAB 2022a platform are discussed in detail. Following this, the proposed methodology results were assessed and compared with those of other existing approaches. The experimental setup for evaluating the performance of the proposed algorithm involves two distinct scenarios based on the placement of BS and the network parameters. In Scenario 1, the BS is positioned at coordinates (150, 150) within a 500 m × 500 m area, with 500 randomly distributed sensor nodes. In Scenario 2, the BS is placed at coordinates (300, 300) within a larger 1000 m × 1000 m area, with 1000 randomly distributed sensor nodes. During each time a node remains in an active state for a single time slot to send, receive, or forward data. For the remaining duration, the node stays in a sleep state to conserve energy. In both scenarios, the sensor nodes are static, and each node is assigned an initial energy of 1 J. To model reliable communication between nodes a transmission success rate is assigned to each link, representing the probability of successful data delivery. The transmission range of the nodes is set to 30 meters, with energy consumption parameters such as transfer power (0.744 nJ/bit/m²), reception power (0.0648 nJ/bit/m²), and idle mode and awake mode power dissipation (0.06 nJ/bit/m² and 0.0175 nJ/bit/m², respectively). The number of CH is configured to 10% or 15% of the total number of nodes, and the number of ZM is set to 0.1% of the total nodes in both scenarios. The network bandwidth is 60 kbps, and the data packet size is 1000 bits, while the control packet size is 200 bits. The simulation runs for 2500 rounds to assess the protocol’s performance. The network’s performance is evaluated based on key metrics including packet reception rate, delay, energy consumption, and network lifetime, and results are compared against other existing protocols to gauge the efficiency of the proposed approach. Table 2 provides an overview of the simulation parameters. Figure 4 Shows the Overview of simulation environment where the nodes are deployed in two scenarios and different network parameters used are presented in this section along with performance metrics considered are also presented. The proposed approach is also compared with approaches like APCLO [27], DPFCP [31], GWOA-CH [29], HOEEACR [34], and EEU-SEC [36]. Evaluation focuses on important performance indicators which are further explained below. These indicators include packet receipt, network longevity, latency, and energy usage

**Table 2:** Network Simulation Metrics and Their Assigned Values.

Network Metrics	Value
Network Bandwidth	60 kbps
Network area size (meters)	500 × 500, 1000 × 1000
Number of Nodes	500, 1000
Size of data packet	1000 bits
Control packet size	200 bits
Sink Node Placement	Scenario 1: x = 150, y = 150
	Scenario 2: x = 300, y = 300
Transmission Range	30 meters
Initial Power	1 J
Transfer Power	0.744 nJ/bit/m^2^
Power of Reception	0.0648 nJ/bit/m^2^
Idle mode power dissipation	0.06 nJ/bit/m^2^
Awake mode power dissipation	0.0175 nJ/bit/m^2^
Number of iterations or rounds	2500 rounds
Node Deployment	Random
Mobility	Static

### Network lifetime comparision of EECR-GEMACHHOA and other algorithms

**Fig 5:**
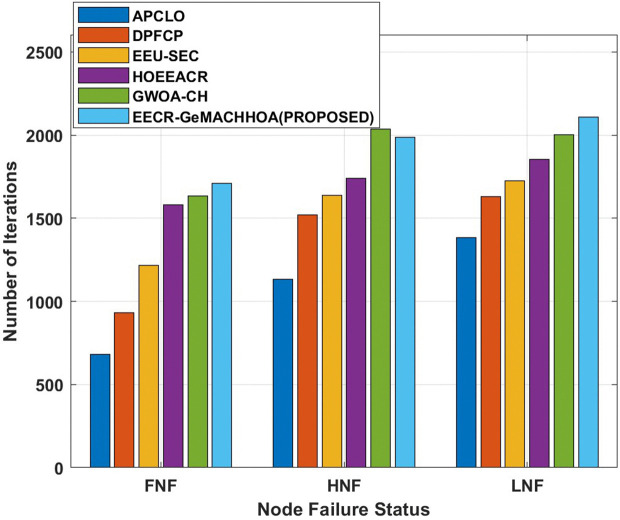
Scenario 1# Failure status vs Iterations(500 nodes 10% CH and 0.1% ZM).

**Fig 6:**
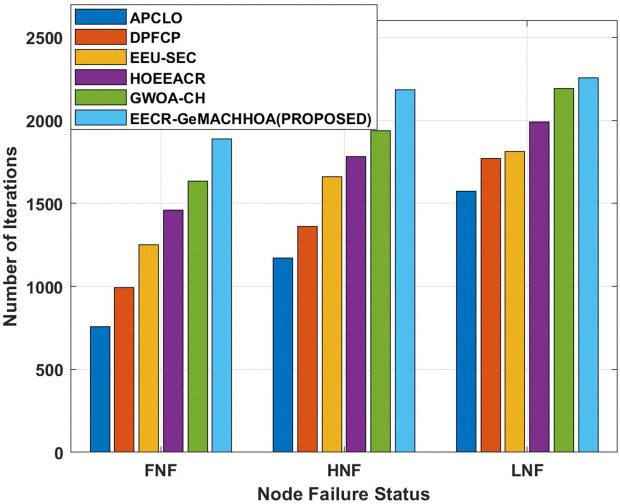
Scenario 2#Failure status vs Iterations (1000 nodes 10% CH and 0.1% ZM).

**Fig 7:**
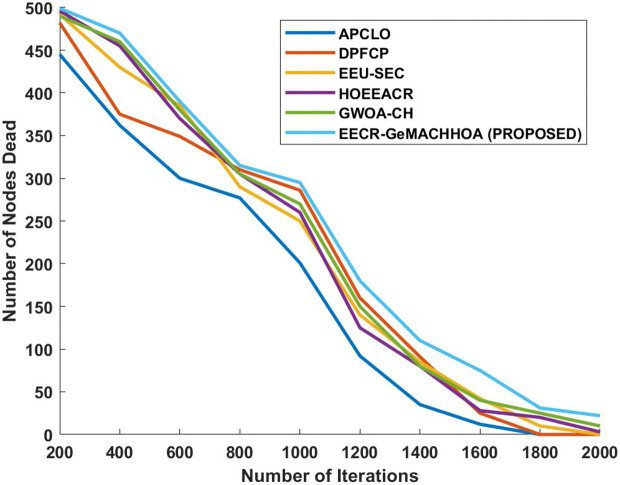
Scenario 1# Number of iterations vs Number of nodes dead (500).

**Fig 8:**
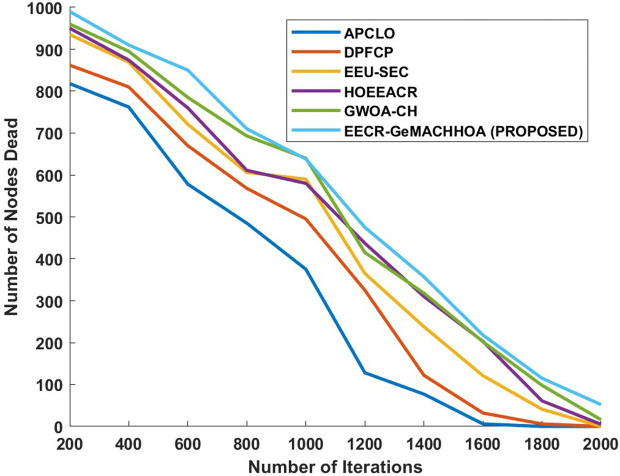
Scenario 2# Number of iterations vs Number of nodes dead (1000).

To compare network lifetimes, simulations measured that a wide variety of nodes remained operational as the number of simulation rounds increased, accounting for nodes that ceased functioning. The proposed EECR-GeMACHHOA method underwent rigorous assessment across diverse node densities and WSN scenarios, always outperforming current algorithms. The network lifespan measures, FNF (first node fail), HNF (half node fail), and LNF (last node fail), are delayed by the proposed model, which is a crucial sign of network stability Fig 5. The nodes that failed over time in terms of rounds based on scenario 1 were limited to 500. EECR-GeMACHHOA achieved a network lifetime of 2186 rounds, surpassing APCLO, DPFCP, EEU-SEC, HOEEACR, and GWOA-CH, which achieved 1382, 1629, 1725,1854 and 2108 rounds, respectively Fig 6. The nodes that failed over time in terms of rounds based on scenario 2 were limited to 1000 nodes. EECR-GeMACHHOA achieved a network lifetime of 2256 rounds, surpassing APCLO, DPFCP, EEU-SEC, HOEEACR, and GWOA-CH, which achieved 1572, 1769, 1812,1991, and 2193 rounds, respectively. EECR-GeMACHHOA integrates critical factors such as transmission range, remaining energy, node degree centrality differences between ZM and CHs, and distances into its fitness functions for ZM and CH selection, additionally for data transfer from ZMs to BS and for multi-hop data transmission between them. Unlike current algorithms that primarily prioritize energy and distance, potentially causing energy imbalances and network hotspots, EECR-GeMACHHOA effectively optimizes energy consumption and extends network lifespan. The analysis of energy intake and network longevity for the duration of the route of the simulation rounds is shown in Fig 7, based on scenario 1 with a node density of 500, which shows that EECR-GeMACHHOA is capable of preserving 21 operational sensor nodes at the end of the simulation Fig 8, based on scenario 2 with a node density of 1000, shows that EECR-GeMACHHOA is capable of preserving 52 operational sensor nodes at the end of the simulation. In contrast, the number of operational nodes maintained by APCLO, DPFCP, EEU-SEC, HOEEACR, and GWOA-CH is less than 20.

### Packet reception comparison of EECR-GEMACHHOA and other algorithms

**Fig 9:**
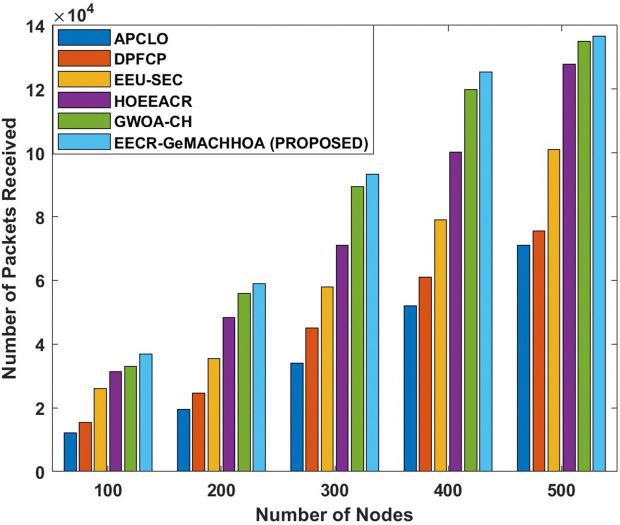
Scenario 1# Packet received with No. of nodes (500).

**Fig 10:**
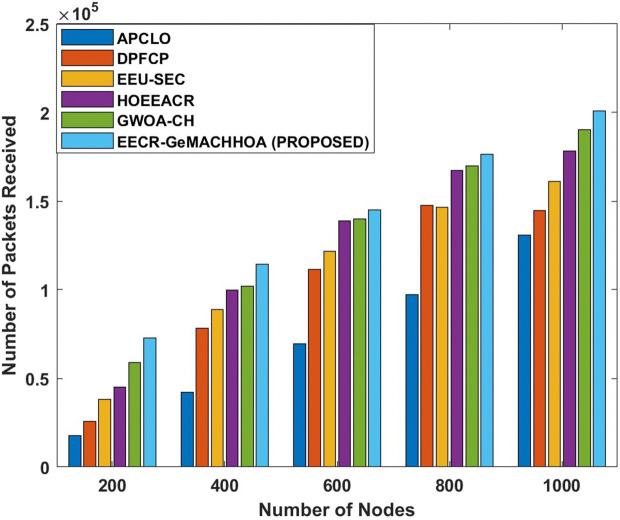
Scenario 2# Packet received with No. of nodes (1000).

Network throughput is an important metric for any type of network because packet loss needs to be reduced, and all packets should reach their destination successfully. Node densities between 200 and 1000 were considered in this study to compare packet reception across multiple algorithms via the suggested EECR-GeMACHHOA method. During simulations, proposed method outperforms protocols such as APCLO, DPFCP, EEU-SEC, HOEEACR, and GWOA-CH. Compared to earlier methods, EECR-GeMACHHOA has longer network durability and reduced energy consumption. Proposed methodology delivers enhanced packet transport by increasing the probability of using dynamic pheromone evaporation methods and ZM, mostly based on translational symmetry toward the BS. Moreover, EECR-GeMACHHOA offers balanced cluster building and successful multi-hop routing by using efficient fitness features to select multiple CHs and ZM. As shown in Fig 9, 10, the method consistently outperforms alternative methods in terms of packet shipping to the bottom station over several rounds, improving by 9.86%. This improvement is credited to the geometrically dynamic pheromone evaporation for selecting the ZM and CH in EECR-GeMACHHOA, which ensures balanced energy distribution among zones and even participant illustration. In scenario 1, with 500 nodes, the proposed method successfully delivered 136600 packets compared to 71048 for APCLO, 79461 for DPFCP, 102119 for EEU-SEC, 127903 for HOEEACR, and 134,914 for GWOA-CH. In scenario 2, with 1000 nodes, EECR-GeMACHHOA delivered 200793 packets, whereas APCLO, DPFCP, EEU-SEC, HOEEACR, and GWOA-CH delivered 130,822, 144,581, 161,285, 178347, and 190314 packets, respectively. These results show that the EECR-GeMACHHOA method achieves sustained energy consumption and improved packet transmission performance to the BS.

### End-to-end delay comparison of EECR-GeMACHHOA and other algorithms

Since WSN employs multi-hop communication to route packets to the BS, the end to end (e2e) latency metric is essential for routing packets should be minimized. The number of forward hops and the media access control (MAC) latency are included in the total routing path delay.

**Fig 11:**
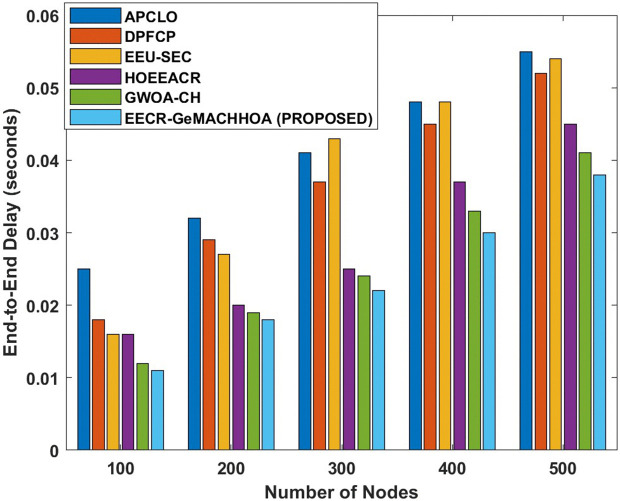
Scenario 1# Latency with No. of nodes (500).

The average e2e latency is shown in Fig 11, 12 together with nodes. With more nodes in the network, the e2e latency rises noticeably, and the differences between the proposed approach and other competing approaches are marginal. For instance, in scenario 1 with 500 nodes, the EECR-GeMACHHOA approach records an e2e delay of 0.038 seconds, while APCLO, DPFCP, EEU-SEC, HOEEACR, and GWOA-CH report delays of 0.055,0.052, 0.048, 0.045, and 0.041 seconds, respectively. Similarly, in scenario 2 with 1000 nodes, the EECR-GeMACHHOA method indicates a delay of 0.049 seconds, which is lower than the delays of 0.059 seconds for APCLO, 0.056 seconds for DPFCP, 0.053 seconds for EEU-SEC, 0.049 seconds for HOEEACR, and 0.045 seconds for GWOA-CH.Thus, the e2e latency needs to be further reduced with additional methods or improved algorithms for transmitting packets via multi-hop communication routing. However, the proposed approach has a minimum delay compared with other approaches. These outcomes demonstrate how well the suggested strategy reduces e2e latency in WSNs.

**Fig 12:**
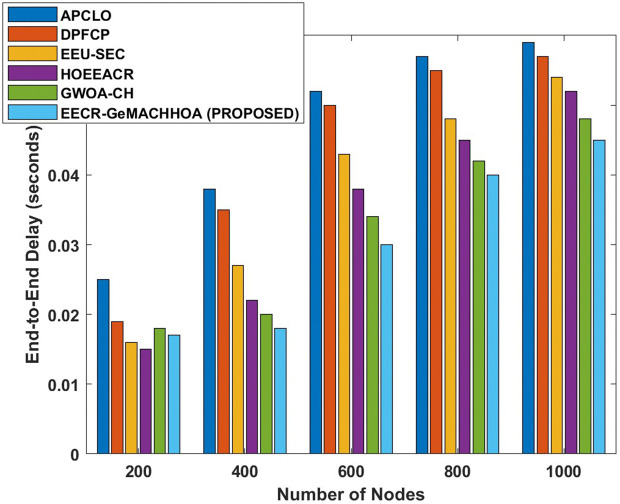
Scenario 2# Latency with No. of nodes (1000).

### Energy consumption comparison Of EECR-GeMACHHOA and other algorithms

**Fig 13:**
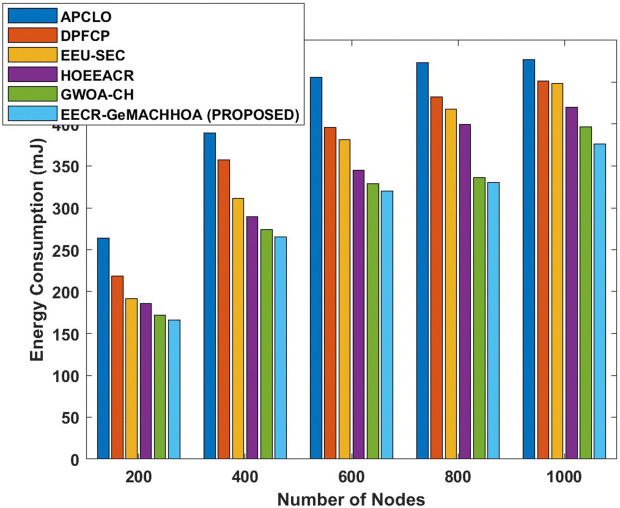
Energy consumption Vs node density.

Energy consumption is an important metric in WSNs since it’s critical to monitor nodes’ and the network’s total residual energy as the number of rounds increases. Data transmission will be effective when network connectivity is maintained by reducing the energy of nodes. EECR-GeMACHHOA optimizes energy consumption across the network by integrating specialized dynamic pheromone evaporation in ant colony with geometric methods for selecting ZMs and CHs. This approach ensures balanced power distribution, which contrasts with previous systems that could lead to uneven energy allocation and network instability due to poorly managed task assignment among CHs and ZMs. EECR-GeMACHHOA strategy enhances overall network stability and sustainability by promoting efficient energy use throughout WSNs. Figure 13 shows the energy intake as a function of node density, with 200–1000 sensor nodes deployed in the region. EECR-GeMACHHOA approach consumes 15.03% less energy than the other techniques as the node density increases. In our scenario with a node density of 1000, the proposed approach consumes 376.15mJ, while APCLO consumes 476.77mJ, DPFCP consumes 451.35mJ, EEU-SEC consumes 448.20mJ, HOEEACR consumes 420.03mJ, and GWOA-CH consumes 397.04mJ. Computer simulations show that the proposed approach surpasses other recent algorithms, including APCLO, DPFCP, EEU-SEC, HOEEACR, and GWOA-CH.

### Statistical analysis

To validate the robustness of proposed EECR-GeMACHHOA approach statistical analysis is conducted over 30 independent simulation runs, reporting the mean, standard deviation, and 95% confidence interval for key metrics such as End-to-End Delay, Energy Consumption, and Packet Delivery Ratio (PDR). The evaluation is compared to existing methods, such as GWOA-CH, APCLO, HOEEACR, and EAHA. The suggested EECR-GeMACHHOA consistently achieves lower delay, lower energy consumption, and higher PDR when compared to all existing models. Existing models’ performance fluctuates but the proposed EECR-GeMACHHOA approach delivers improvements that are not only significant but also statistically reliable. The statistical analysis validates its suitability for border surveillance where both efficiency and consistency are critical.

#### End-to-end delay analysis.

A lower delay indicates faster data transmission, which enhances the timeliness and reliability of the network. Table 3 represents the comparative delay performance across existing models and the proposed EECR-GeMACHHOA approach. Results shows that APCLO experiences the highest delay (0.042 s) due to its conventional hierarchical clustering and lack of adaptive optimization. DPFCP reduces delay to 0.039 s by applying adaptive power control, but still suffers from uneven load distribution. EEU-SEC improves delay (0.037 s) and HOEEACR further improves delay (0.036 s) by integrating hybrid optimization strategies, while GWOA-CH achieves 0.034 s through bio-inspired CH selection using Grey Wolf Optimization.

**Table 3:** End-to-end delay statistical analysis (n = 30 runs).

Algorithm	Mean Delay (s)	Std. Dev.	95% CI (±)
APCLO	0.042	0.0028	0.00105
DPFCP	0.039	0.0027	0.00101
EEU-SEC	0.037	0.0026	0.00097
HOEEACR	0.036	0.0025	0.00093
GWOA-CH	0.034	0.0020	0.00075
Proposed (EECR-GeMACHHOA)	0.029	0.0018	0.00067

In contrast, the proposed EECR-GeMACHHOA achieves the lowest delay of 0.029 s, primarily because of its hybrid framework that optimizes zone head, CH selection and routing paths simultaneously. Smaller standard deviation (0.0018) and narrower 95% confidence interval (±0.00067) confirm that the proposed approach not only reduces delay but also ensures stable and consistent performance across multiple simulation runs. This demonstrates the robustness of EECR-GeMACHHOA in minimizing transmission latency while maintaining reliability under stochastic variations.

#### Energy consumption analysis.

Minimizing energy consumption directly extends the overall network lifetime, which is essential for applications such as environmental monitoring and border surveillance. Table 4 presents the comparative energy consumption results across different existing algorithms. APCLO records the highest average energy consumption (312.45 mJ) due to its conventional clustering and lack of adaptive optimization mechanisms. DPFCP lowers consumption to 291.67 mJ by introducing adaptive power control, yet it still suffers from imbalanced load distribution. EEU-SEC improved consumption (271.82 mJ) and HOEEACR achieves further improvement (268.39 mJ) by integrating hybrid clustering and routing strategies, while GWOA-CH reduces energy consumption to 251.18 mJ using Grey Wolf Optimization for efficient CH selection. In contrast, the proposed EECR-GeMACHHOA consumes only 204.76 mJ, representing a significant reduction compared to existing approaches. Furthermore, the lower standard deviation (3.1) and narrower confidence interval (±1.14) indicate that the proposed method not only reduces energy usage but also maintains consistency across multiple simulation runs. These results validate the effectiveness of EECR-GeMACHHOA in enhancing energy efficiency, thereby contributing to a prolonged network lifetime and more sustainable WSN operations.

**Table 4:** Energy consumption statistical analysis (n = 30 runs).

Algorithm	Mean Energy (mJ)	Std. Dev.	95% CI (±)
APCLO	312.45	4.8	1.75
DPFCP	291.67	4.5	1.65
EEU-SEC	271.82	4.4	1.60
HOEEACR	268.39	4.3	1.57
GWOA-CH	251.18	3.9	1.43
Proposed (EECR-GeMACHHOA)	204.76	3.1	1.14

#### Packet delivery ratio analysis.

Packet Delivery Ratio (PDR) is one of the most important performance indicators in WSNs, as it measures the percentage of successfully delivered packets over the total packets generated in the network. Table 5 summarizes the PDR performance of existing algorithms. APCLO achieves the lowest PDR (91.12%), primarily because its conventional hierarchical routing does not incorporate with adaptive load balancing, making it more prone to packet drops. DPFCP improves the ratio to 92.89% by applying adaptive power control, but uneven traffic distribution still leads to packet losses under high load. EEU-SEC further enhances PDR to 93.01% and HOEEACR further enhances PDR to 93.07% through hybrid clustering and routing optimization, while GWOA-CH reaches 94.19% by leveraging Grey Wolf Optimization for efficient CH selection and balanced communication paths. The proposed EECR-GeMACHHOA achieves the highest PDR of 95.72%, benefiting from hybrid combination for effective ZM, CH selection and energy-aware routing. This integrated mechanism not only improves delivery efficiency but also effectively mitigates packet drops caused by congestion. Furthermore, the low standard deviation (0.39) and confidence interval (±0.14) confirm that the proposed model consistently maintains high reliability across multiple simulation runs.

**Table 5:** Packet Delivery Ratio (PDR) statistical analysis (n = 30 runs).

Algorithm	Mean PDR (%)	Std. Dev.	95% CI (±)
APCLO	91.12	0.52	0.19
DPFCP	92.89	0.50	0.18
EEU-SEC	93.01	0.49	0.18
HOEEACR	93.07	0.48	0.18
GWOA-CH	94.19	0.45	0.17
Proposed (EECR-GeMACHHOA)	95.72	0.39	0.14

## Discussions

The EECR-GeMACHHOA algorithm, which integrates dynamic pheromone evaporation, and HHO for CH selection and routing achieves superior performance by addressing key challenges in network optimization, such as energy efficiency, scalability, and robustness. Energy efficiency is a critical factor since nodes are often battery-powered, and energy depletion leads to network failures. The proposed method optimizes energy consumption in several ways, the pheromone updates in the ACO model allow ants to select CHs and optimal paths based on node residual energy, thereby favoring energy-efficient nodes. Dynamic pheromone evaporation further adjusts the pheromone levels, making sure that nodes with higher residual energy remain favored for CH selection by considering $RES_{ht}$ (normalized residual energy) as a factor in fitness function (Eq. 26), the algorithm ensures that energy is distributed more evenly, preventing premature exhaustion of any single node. The multi-hop routing mechanism reduces the energy cost for nodes by ensuring that long-range communication is distributed over multiple nodes, minimizing the need for high-power transmissions. Scalability is an important feature for WSNs, particularly as the network size increases.

Zone-based clustering divides the network into zones and selecting CHs for each zone independently, the algorithm reduces the overhead associated with centralized control and ensures that the network can scale efficiently as more zones are added. The ACO-based method allows each node to autonomously contribute to the network’s organization, and HHO dynamically adapts to changes in the network topology (e.g., node failures or additions), making it scalable under various conditions. The exploration phase (Eq. 31) ensures that the algorithm searches for a wide range of possible paths to the BS, while the exploitation phase (Eqs. 32–36) fine-tunes these paths to ensure optimal energy efficiency and low latency. The HHO’s flexibility in switching between exploration and exploitation ensures that the algorithm can find reliable paths even under changing network conditions by incorporating factors such as energy efficiency and node connectivity, the algorithm promotes a longer network lifetime.

From the statistical results, it is evident that the proposed EECR-GeMACHHOA outperforms existing algorithms in all three performance metrics. The lower mean values, reduced standard deviations, and tighter confidence intervals demonstrate not only superior performance but also higher consistency. This makes the proposed model particularly well-suited for large scale sensitive WSN applications such as border surveillance.

## Conclusion & opportunities of future research

This study suggests an energy efficient EECR-GeMACHHOA optimized routing method with optimal ZM and CH selection. In order to optimize network performance, this strategy is implemented in MATLAB 2022 and three performance indicators were utilized includes residual energy, node density, and energy consumption rate to compare the outcomes with those of APCLO, DPFCP, EEU-SEC, HOEEACR, and GWOA-CH. The selection of ZM and CH, depending on residual energy and connection, further guarantees that the routing pathways are resilient and reduce energy depletion across the network. The algorithm’s ability to adaptively adjust based on the network conditions provides a significant improvement over traditional routing protocol. The simulation results show that the proposed approach performed better than existing approaches.

Future research will include incorporating environmental elements, which includes sink node mobility, multiple sink node deployment and ranging signal energy, into the algorithm to make it more adaptable to real global conditions in which node positions and network topology can change over time. This approach might enhance the robustness and applicability in diverse deployment scenarios. Hybrid algorithms such as parallelizing ACO and HHO computations can leverage the strength of multiple optimization techniques, leading to more efficient and powerful routing solutions. Exploring those directions can similarly enhance the overall performance, reliability, and scalability, making it more suitable for complicated and dynamic WSN environments.
